# Coronaviruses in Bats: A Review for the Americas

**DOI:** 10.3390/v13071226

**Published:** 2021-06-25

**Authors:** Itandehui Hernández-Aguilar, Consuelo Lorenzo, Antonio Santos-Moreno, Eduardo J. Naranjo, Darío Navarrete-Gutiérrez

**Affiliations:** 1 El Colegio de la Frontera Sur, Unidad San Cristóbal, San Cristóbal de Las Casas 29290, Chiapas, Mexico; clorenzo@ecosur.mx (C.L.); enaranjo@ecosur.mx (E.J.N.); dnavarre@ecosur.mx (D.N.-G.); 2 Centro Interdisciplinario de Investigación para el Desarrollo Integral Regional, Unidad Oaxaca, Instituto Politécnico Nacional, Santa Cruz Xoxocotlán 71230, Oaxaca, Mexico; asantosm90@hotmail.com

**Keywords:** *Alphacoronavirus*, *Betacoronavirus*, Chiropterans, COVID-19, host, MERS, SARS

## Abstract

The SARS-CoV-2 coronavirus is the focus of attention as it has caused more than three million human deaths globally. This and other coronaviruses, such as MERS-CoV, have been suggested to be related to coronaviruses that are hosted in bats. This work shows, through a bibliographic review, the frequency of detection of coronavirus in bats species of the Americas. The presence of coronavirus in bats has been examined in 25 investigations in 11 countries of the Americas between 2007 and 2020. Coronaviruses have been explored in 9371 individuals from 160 species of bats, and 187 coronavirus sequences have been deposited in GenBank distributed in 43 species of bats. While 91% of the coronaviruses sequences identified infect a single species of bat, the remainder show a change of host, dominating the intragenera change. So far, only Mex-CoV-6 is related to MERS-CoV, a coronavirus pathogenic for humans, so further coronavirus research effort in yet unexplored bat species is warranted.

## 1. Introduction

Bats have been involved in at least three instances of coronavirus spillover to humans, most recently SARS-CoV-2, the cause of the coronavirus disease COVID-19 and the current pandemic. This virus emerged in late December 2019 in the province of Wuhan, China, and has infected more than 178 million people worldwide, resulting in more than three million deaths [1,2]. However, since 2002 there have been two precedents for highly pathogenic coronaviruses for humans. The first, the SARS-CoV coronavirus, emerged in Guangdong, China, and is the cause of severe acute respiratory syndrome (SARS), which infected more than 8000 people and caused 774 deaths worldwide, for which it was considered the first pandemic of the 21st century [3,4]. Ten years later, in 2012, the MERS-CoV virus that causes Middle East respiratory syndrome emerged in Saudi Arabia. This virus had more than 2000 cases and 803 deaths in humans in 27 countries [5,6].

The probable source of the coronavirus emergence has been proposed to be related to other genetically similar viruses detected in bats. However, scientific evidence indicates that the SARS-CoV, MERS-CoV, and SARS-COV-2 coronaviruses could probably jump to humans through other intermediate hosts, such as palm civets (*Paguma larvata*), raccoon dogs (*Nyctereutes procyonoides*), and camels (*Camelus dromedarius*), to infect humans [7,8,9]. Thus, human coronaviruses are considered to have a zoonotic origin [10,11,12].

Coronaviruses are single-stranded positive-sense RNA viruses with genomes of 16 to 31 kb, belonging to the order of Nidovirales and to the Coronaviridae family, which is subdivided into the *Letovirinae* and *Orthocoronavirinae* subfamilies [12,13,14]. The latter includes the genera *Alphacoronavirus* (α-CoV); *Betacoronavirus* (β-CoV), containing coronaviruses related to MERS-CoV, SARS-CoV, and SARS-CoV-2; *Gammacoronavirus* (γ-CoV); and *Deltacoronavirus* (δ-CoV). The first two are found in mammals, including bats, while the latter two have been recorded mainly in wild and domestic birds [12,13,14].

Bats are the group of mammals that harbor the largest number of coronavirus species [14]. As of 2019, more than 200 coronaviruses had been identified in bats from Asia, Africa, Europe, and the Americas. Recent estimates suggest there could be at least 3204 coronaviruses in bats, so there are many yet to be discovered [15]. Initially, the research effort to discover coronaviruses harbored by bats was focused on China (e.g., [16,17,18]), Asia, Europe, and Africa (e.g., [11,19,20]). Less research effort has been expended in the New World, so our knowledge about the diversity of coronaviruses and the host species remains incomplete [21,22].

In this work, we review the available studies on natural infection (detection of viral RNA in samples of bats captured in their natural habitat) and experimental infection (coronavirus infection in bats carried out in the laboratory, adaptation, and replication of coronavirus, and virus isolation) of coronavirus in bats in the Americas up to 2020. We compiled a list of coronavirus host species and the number of positive individuals and showed the coronavirus-host patterns that predominate in the Americas. The results of this study contribute to forming a baseline to identify the information gaps in matters of zoonotic disease management and pandemic control.

## 2. Materials and Methods

A review of the existing literature on bat and coronavirus studies in the Americas was conducted through a systematic search in Google Scholar, Web of Science, PubMed, NCBI (National Center Biotechnology Information), and PMC (National Library of Medicine). We also searched the world databases of viruses associated with bats (http://www.mgc.Ac.cn/DBatVir/; accessed on 19 September 2020) and mammals compiled by [23] (http://doi.org/10.5281/zenodo.596810; accessed on 19 September 2020). The search included the following words in Spanish and English: *Alphacoronavirus*, America, *Betacoronavirus*, Coronaviridae, coronavirus, CoV, host, MERS, bats, pandemic, chiroptera, reservoir, SARS, viroma, and zoonosis. Only works that addressed natural and experimental infection were considered for this study. Studies that did not present the taxonomy of the species and the number of individuals examined were not considered for the analysis.

Using the data available for each individual and species in the published studies, a database was built that included the following fields: species, family, genera, year and month of collection, country, state, coordinates, collection site, detection method, organs analyzed, coronavirus sequence detected, coronavirus sequence accession number in GenBank, result (positive or negative for coronavirus), and bibliographic reference. All sequence accession numbers were corroborated in the GenBank genetic sequence database, which helped fill in information not available in the articles. A map of the American continent was prepared in the ArcGis Desktop [24] version 10.2.1 software program showing the countries where coronavirus studies have been carried out in bats, as well as the host bat species.

A presence/absence matrix of the viral sequences and the coronavirus host bat species was constructed. From this matrix, a bipartite network made up of nodes and edges or links was generated, which allows graphically observing the interaction between two groups [25], in this case, the host bat species and the coronaviruses sequences that have been identified in each species. It also allows the calculation of indices that summarize the structure of the network, such as connectivity (real number of interaction compared to the number of possibilities given the size of the matrix), asymmetry (degree of interaction between coronavirus sequences and bats), nesting (hierarchical ordering of nodes, where the most specialized have interactions with the generalists), robustness (assesses the stability of the network), generality (for the higher level, i.e., the host species) or vulnerability (for the lower level, i.e., coronaviruses), and specialization (H2, indicates how diverse the interactions are). All analyses were performed with the bipartite package [25] in the RStudio program [26].

## 3. Results

### 3.1. Current State of Knowledge

**Figure 1:**
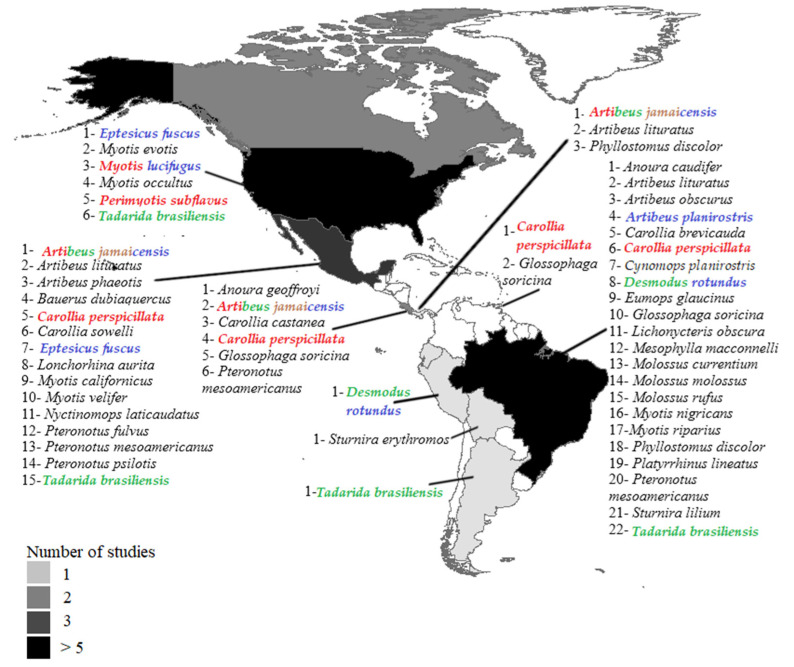
Geographic location of the countries of the Americas where coronavirus studies have been carried out in bats. The list of bat species (naturally infected) with coronavirus sequences deposited in GenBank is indicated for some countries. The names of the bat species in which experimental infection has been carried out are shown in different colors. Red: cell infection; green: viral isolation; brown: seroconversion; purple: coronavirus adaptation.

**Figure 2:**
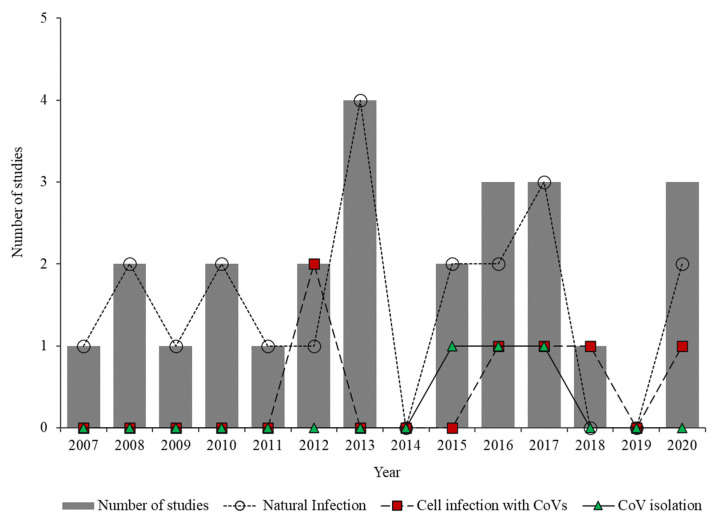
Number of studies examining natural infection, experimental coronavirus infection in bat cells, and coronavirus isolation over time.

The bibliographic search yielded 25 articles published between 2007 and 2020 in 11 countries of the Americas (76% in the decade from 2011 to 2020) (Figure 1). Countries where research was conducted (the number of studies is in parentheses; percentage indicates total positive individuals) include Brazil (9; 29.3%), United States (6; 25.6%), Mexico (3; 17.1%), Canada (2; 14.9%), and Costa Rica (2; 9.6%). One study each was conducted in Panama (1.9%), Bolivia (0.5%), Trinidad and Tobago (0.5%), Argentina (0.3%), and Peru (0.3%) (Figure 1). Of the 25 published articles, 68% examined natural coronavirus infection in bats, 16% experimental coronavirus infection in bat cells, 8% both natural infection and experimental infection, and 8% both natural infection and coronavirus isolation (Figure 2).

### 3.2. Coronavirus Host Bats

Information on the presence of coronavirus was investigated in 9731 bat individuals (Table 1). Of these, 9238 individuals (98.58%) were reported at the taxonomic level of species and 133 (1.41%) to genera. Prior to 2020, natural coronavirus infection had been explored in 160 species of bats in the Americas; available coronavirus sequences were found in GenBank in 43 species (346 individuals) (Table 1) in 10 countries (Figure 1). Of the 43 species, 46.51% belong to the *Phyllostomidae* family, 27.90% to *Vespertilionidae*, 18.60% to *Molossidae,* and 6.97% to *Mormoopidae*. Experimental infection had been carried out in nine species of bats with the *Alphacoronavirus* hCoV-NL63 and the *Betacoronavirus* MERS-CoV and hCoV-EMC, coronaviruses that mainly affect humans (Table 2). The first records of individuals positive for coronavirus by natural infection in the Americas were in 2007 in *Eptesicus fuscus* and *Myotis occultus* [27]. The species with the highest number of coronavirus-positive individuals were *Eptesicus fuscus* (21.1%, *n* = 73) and *Myotis lucifugus* (16.5%, *n* = 57). Of the 160 species that have been examined over time, in 70 (43.8%) only 1 to 5 individuals were examined, while in 21 species (13.1%) more than 100 individuals were evaluated (Table 1).

**Figure 3:**
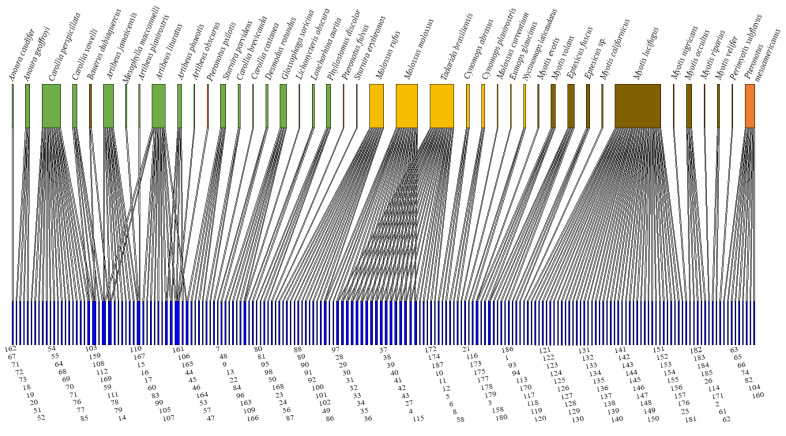
Bipartite network of coronaviruses sequences available in GenBank detected in bat species in the Americas. The upper boxes are the host bat species that belong to the *Phyllostomidae* family (green), *Molossidae* (yellow), *Vespertilionidae* (brown), and *Mormoopidae* (orange). The lower boxes in blue correspond to the coronavirus viral sequence according to the coronavirus sequence number in Table S1.

Altogether, 187 coronavirus viral sequences were described for 43 species of bats (Figure 3, Supplementary Material 1). The coronavirus sequence was not described for one species, *Platyrrhinus lineatus.* Of the 151 coronaviruses described at the genera level, 89.4% were *Alphacoronavirus* and 10.6% *Betacoronavirus*. The 34.8% of the coronavirus sequences available in GenBank have been detected in bat species of the *Vespertilionidae* family, followed by *Phyllostomidae* (33.2%), *Molossidae* (25.1%), and *Mormoopidae* (5.4%) (Supplementary Material 1). Bats from more than one family represented 1.6% of infections. The majority (91%) of coronaviruses sequences have been detected in a single species of bat, while 8.9% of coronaviruses sequences have been detected in more than one species of bat that is, they showed a change of host (Figure 3). The *Phyllostomidae* family was associated with 60% of the coronaviruses sequences that presented a change of host.

To date, the 43 coronavirus-positive bat species harbored an average of 7.5 coronaviruses sequences, with the highest values recorded in *Myotis lucifugus* (*n* = 42), *Tadarida brasiliensis* (*n* = 39), and *Molossus molossus* (*n* = 38), while in 10 bat species only one coronavirus sequence was recorded. The network shows a low connection (0.02) with a moderate asymmetry (0.6), and a low nesting of the matrix (2.3). Further, robustness is moderate (0.5), vulnerability low, and 16.5 species of bats per coronavirus sequence. Specialization is zero (0.0), indicating a high degree of generalization (0.16).

## 4. Discussion

### 4.1. Current State of Knowledge

Since the SARS pandemic in China in 2002, and after the first report of coronavirus in bats *Miniopterus pusillus* in Hong Kong [50], the number of studies on bats and coronaviruses has increased in various regions of the world, including the Americas [51,52]. Since then, many coronaviruses have been described in different bat families (e.g., [15,20,27]). Furthermore, the outbreak of MERS in 2012 in China, and the description of the coronavirus Mex-CoV-6 in the molosid bat *Nyctinomops laticaudatus* from Mexico, which is phylogenetically related to MERS-CoV [21], increased the relevance of researching and disseminating knowledge about the coronaviruses of bats in the Americas.

Advances in molecular techniques have made it possible not only to identify the coronavirus host species but also to determine the mechanisms by which bats allow or restrict the replication of these viruses [53]. For example, between 2007 and 2011, studies in the Americas focused on detecting natural coronavirus infection; later, in 2012, studies were conducted that detected the natural and experimental infection of coronavirus in bat cells; and since 2015 attempts have been made to isolate these viruses from bat cells (Figure 2). Despite these efforts, until 2020 this type of study was lacking in 70% of the countries of the Americas. Therefore, and because it has been suggested that Latin America is a focus of emerging infectious diseases [54], it is necessary to carry out a greater number of studies, especially in countries of the Americas where such studies are lacking to date.

### 4.2. Coronavirus Host Bats

The 160 species of bats in which coronaviruses have been tested represent more than 45% of the more than 350 species that are distributed in the Americas and the Caribbean [55]. In general, a positivity percentage of 3.7% was obtained. However, in each study of natural infection the prevalence ranged between 0.9% [31] and 33.3% [45]. Coronavirus prevalence rates in bats have been reported to typically range from 3% to 10% [30], and a global study with more than 12,000 samples reported a rate of 8.6% [15]. Such estimates may be influenced by factors such as the geographic regions involved, the number of species analyzed in each study, the heterogeneity of the collection sites, the type of samples analyzed, and the number of individuals sampled [15,30]. This review found that only three of the 25 studies examined samples from more than one country, and studies that examined a greater number of species also recorded a greater number of species positive for coronavirus. In this study we found that of the 43 species of bats with coronavirus sequences available in GenBank, 46.51% belong to the *Phyllostomidae* family and 27.90% to *Vespertilionidae*. This may be related to the fact that they are the families with the highest number of species in the Chiroptera order and are best studied in ecological studies. For example, of the 160 species considered so far, more than 70% are species of the *Phyllostomidae* and *Vespertilionidae* family and only the *Artibeus*, *Carollia,* and *Sturnira* genera represent more than 43% of all individuals, which, contains abundant species in the *Neotropics* both in conserved habitats and urban areas, in addition to being frequent in captures with fog nets, a method reported with greater frequency in the studies of this research [56].

Regarding the type of samples analyzed, the results show that coronaviruses have a positive tropism toward the intestinal tract, as they are detected in a higher percentage in fecal samples (43.1%) and intestines (35.1%) compared to liver tissue samples, lung, spleen, and kidney (20.3%), saliva (1.2%) or blood (0.3%). Intestinal tropism has been reported in previous studies in other geographic areas [14,17,57]. Therefore, bat excretions could be the main source of coronaviruses and a relevant factor in spillover events for the transmission of coronavirus to humans through contact between the susceptible host and the excretions or articles contaminated with them [14].

In relation to the specimens sampled, of the 160 evaluated species that this study compiled, in 43.8% of them the number of individuals examined was less than five, which could have limited the probability of detecting at least one coronavirus. However, even though in *Lichonycteris obscura*, *Lonchorhina aurita*, *Cynomops planirostris,* and *Myotis riparius* the number of individuals examined was very low (2, 4, 5, and 5, respectively), a coronavirus was identified. In contrast, in other species such as *Saccopteryx bilineata*, *Sturnira hondurensis,* and *Phyllostomus hastatus* for which more than 100 individuals were examined, none were positive for coronavirus. These varying rates indicate that some species of bats may be more susceptible to coronavirus infection than others. According to recent estimates, future studies should consider and include fecal samples from 154 to 400 individuals per species to maximize the possibility of detecting all the coronaviruses that a species harbors [15]. However, doing so may not be feasible for rare species or those in some risk categories, such as *Leptonycteris nivalis* and *Myotis lucifugus* (classified as endangered according to the IUCN) [58].

The factors that could favor the natural infection of coronavirus in bats have been little addressed, but it has been suggested that juvenile individuals or lactating females captured in the dry season of the year are more likely to be positive for coronavirus [15,35,59]. In this review, we observed that the ecological data of the evaluated species was absent for most of the individuals, and only data for age and sex were obtained for 1.2% and 4.7% of the individuals, respectively. Therefore, more detailed information should be collected on the captured specimens, such as age, sex, month of capture, and reproductive condition, to allow evaluation of the factors that promote or facilitate infection in bats.

The results obtained from the bipartite network showed moderate asymmetry (0.6) and low connectivity (0.02). This may be because the number of coronavirus sequences nodes was four times greater than the number of nodes of the host bat species, and therefore nesting was also low (2.3) since specialist coronaviruses those that have been detected in a single species of bat—have little interaction with generalist bats species of bats which have more than one different sequence of coronavirus. The bipartite network of this review showed that bats that have been studied in the Americas can host up to 7.5 coronaviruses sequences on average. Furthermore, considering that 75% of the living genera of bats are found in the Americas [60], greater surveillance of coronavirus should be promoted in bat species that have not yet been evaluated or that present a low number of individuals examined (see Table 1), which would help detect unknown coronaviruses or coronaviruses related to known pathogens.

Until 2020, only one of the viral sequences, Mex-CoV-6, which infects *Nyctinomops laticaudatus* in Mexico, was closely related to MERS-CoV, a virus that is pathogenic for humans. The foregoing places Mexico as a potential focus of emerging diseases, although since only one of the 187 viral sequences described so far presents this relationship, a low risk of spillover from coronavirus pathogens for humans is suggested in the Americas [21,30,31]. However, it is important to note that in experiments conducted in the Americas prior to 2020 that examined the infection of bat cells with pathogenic coronaviruses such as MERS-CoV, HCoV-EMC, and HCoV-NL63, there appeared to be no restrictions for entry of coronavirus, and in some cases, viruses successfully replicated for up to nine days [40,46,47,48].

In prior studies, none of the individuals naturally infected by coronavirus presented signs of disease. Most of the individuals infected in laboratory experiments also did not show obvious clinical manifestations of the disease, except for *Artibeus jamaicensis*, which presented histopathological changes in lung cells [46]. Ref. [35] showed, through individuals recaptured more than once in the United States, that bats do not remain persistently positive for coronavirus, and that they could experience temporary infections of up to four months during hibernation without showing signs of disease [43]. Research to determine the mechanisms by which bats limit the disease or develop an immune response at the gene, cell, or in vivo level is a relatively recent field and difficult to carry out due to high costs, lack of inputs, and little success in the development of bat cell lines [53,61].

Coronavirus research in bats around the world will continue to be important to characterize and understand the circulation of coronaviruses to identify potential sources of spread or spillover into the human population [30]. Recent phylogenetic data show that the SARS-CoV-2 coronavirus, which has caused the current COVID-19 pandemic, is not closely related to some Mexican bat coronaviruses (61). However, it is urgent to examine the phylogenetic closeness of SARS-CoV-2 with the rest of the bat coronavirus species and to help clarify the potential to infect humans or other wildlife species and to spread on a large scale, since the ongoing COVID-19 pandemic has demonstrated that advances in knowledge are needed to predict future viral outbreaks [61,62]. Studies like this one aim to improve our understanding of the coronaviruses that bats harbor, not to focus attention on them to incite their slaughter in retaliation for the coronaviruses they harbor.

## Figures and Tables

**Table 1:** Bat species in which natural coronavirus infection has been explored in the Americas.

Bat Species	N ^†^	NP ^‡^	%	CS ^§^	Country ^¶^	Samples Examined ^#^	CoV ^††^	Reference
*Emballonuridae*								
*Balantiopterix plicata*	8	0	0	Up	M	T		[28]
*Cormura brevirostris*	1	0	0	Up	Br	S,U,R,T		[15]
*Peropteryx kappleri*	5	0	0		Cr	F		[29]
*Peropteryx leucoptera*	1	0	0	Up	Br	T		[30]
*Rhynchonycteris naso*	6	0	0	Up	Br,E,M	F,S,U,R,T		[15,21,29]
*Saccopteryx bilineata*	122	0	0	Up	Br,Cr,M,Pa	F,S,U,R,T		[15,21,29,31]
*Saccopteryx canescens*	1	0	0	Up	Br	S,U,R,T		[15]
*Saccopteryx gymnura*	1	0	0	Up	Br	S,U,R,T		[15]
*Saccopteryx leptura*	2	0	0	Up	Br,Pa	F,S,U,R,T		[15,29]
*Saccopteryx* sp.	1	0	0	Up	Br	S,U,R,T		[15]
*Molossidae*								
* *Cynomops abrasus*	11	3	27.2		Br	F,T	α	[32]
* *Cynomops planirostris*	5	3	60		Br	F,T	α	[32]
* *Eumops glaucinus*	41	1	2.4	Up	Br,Cr	F,R,T	β	[28,30,31,33]
*Eumops maurus*	1	0	0		E	F		[29]
*Eumops perotis*	1	0	0		Br	T		[28]
*Eumops* sp.	7	0	0	Up	Br	S,U,R,T		[15]
*Molossops temminckii*	1	0	0	Up	Br	T		[30]
* *Molossus currentium*	38	1	2.6	Up	B,Br,	S,U,R,T	α	[15,29]
*Molossus major*	25	0	0		T	G,R		[28]
* *Molossus molossus*	239	3	1.2	Up	B,Br,Pa	F,S,U,R,T	α	[15,28,29,30,33,34]
* *Molossus rufus*	119	9	7.5	Up	B,Br,Cr,E	F,S,U,R,T	α	[15,28,29,30,31,33]
*Molossus sinaloae*	65	0	0	A,Up	Cr	F,R		[31]
* *Nyctinomops laticaudatus*	10	2	20	Cv,Up	M,Br	B,F,S,U,R,T	β	[15,21,30]
*Nyctinomops macrotis*	45	0	0	Up	M	B,F,S,U,R,T		[15,21]
*Promops nasutus*	1	0	0	Up	B	S,U,R,T		[15]
* *Tadarida brasiliensis*	265	20	7.5	Cv,Up	A,B,Br,U,M	B,G,F,S,U,R,T	α	[15,21,27,33,34,35,36,37,38,39]
*Natalidae*								
*Natalus lanatus*	5	0	0		Cr	F		[29]
*Natalus mexicanus*	34	0	0	Up	M	S,U,R,T		[15]
*Vespertilionidae*								
*Antrozous pallidus*	13	0	0	P	U	F		[35]
*Dasypterus ega*	1	0	0	Up	B	S,U,R,T		[15]
*Histiotus velatus*	24	0	0	Up	B	S,U,R,T		[15]
* *Bauerus dubiaquercus*	42	2	4.7	Up	M	S,U,R,T		[15,21]
*Corynorhinus mexicanus*	13	0	0	Up	M	S,U,R,T		[15,21]
*Corynorhinus townsendii*	4	0	0	Up	U	F		[35]
*Eptesicus andinus*	2	0	0	Up	B	S,U,R,T		[15]
*Eptesicus brasilensis*	10	0	0	A	Cr	F,R		[31]
*Eptesicus furinalis*	13	0	0	A,Up	Br,Cr,M	F,S,U,R,T		[15,30,31,33]
* *Eptesicus fuscus*	647	73	11.1	A,Cv,Up	Cr,U	F,S,R,T	α	[21,27,31,35,40,41]
* *Eptesicus* sp.	1	1	100	Cv,Up	Br,M	B,T	α	[21,30]
*Euderma maculatum*	3	0	0	P	U	F		[35]
*Lasionycteris noctivagans*	44	0	0	Up	U	F,S,R		[27,35]
*Lasiurus cinereus*	47	0	0	Up	Br,Ca,U,M	F,T		[28,33,35,42]
*Lasiurus ega*	19	0	0	Up	B	S,U,R,T		[15]
*Lasiurus intermedius*	2	0	0	Up	M	S,U,R,T		[15,21]
*Myotis albescens*	10	0	0	Up	B,Br,E	F,S,U,R,T		[15,29]
* *Myotis californicus*	11	1	9.0	Up	U,M	F,S,U,R,T		[15,35]
*Myotis ciliolabrum*	30	0	0	Up	U	F,T		[27,35]
*Myotis elegans*	1	0	0	A	Cr	F,R		[31]
* *Myotis evotis*	59	1	1.6	Cv,Up	U	F,S,R,T		[27,35]
*Myotis keaysi*	37	0	0	Cv,Up	B,M	S,U,R,T		[15,21]
*Myotis levis*	1	0	0	Up	Br	S,U,R,T		[15]
* *Myotis lucifugus*	243	57	23.4	C,Up	Ca, U	F,T	α	[35,41,42,43]
* *Myotis nigricans*	55	1	1.8	A,Up	B,Br,Cr,E,M,Pa,Pe	F,S,U,R,T		[15,21,28,29,30,31,33]
* *Myotis occultus*	40	5	12.5	Up	U,M	B,F,S,U,R,T	α	[15,21,27,35]
*Myotis oxyotus*	53	0	0	Up	B	S,U,R,T		[15]
* *Myotis riparius*	5	1	20	Up	B,Br	S,U,R,T	α	[15,33]
*Myotis* sp.	22	0	0	Up	B,Br	S,U,R,T		[15]
*Myotis thysanodes*	22	0	0	Cv	U	F		[35]
* *Myotis velifer*	24	6	25	Up	M	B,F,S,U,R,T	α	[15,21]
* *Myotis volans*	154	12	7.8	Cv,Up	U	F,S,R,T	α	[27,35]
*Myotis yumanensis*	18	0	0	Cv	U	F		[35]
*Parastrellus hesperus*	14	0	0	Cv	U	F		[35]
* *Perimyotis subflavus*	6	2	33.3	Up	U	F		[40,41]
*Rhogeessa io*	3	0	0	A,Up	B,Cr	F,S,U,R,T		[15,31]
*Rhogeessa tumida*	6	0	0	Cv	Cr,Pa	F,R		[29,31]
*Mormoopidae*								
*Mormoops megalophylla*	32	0	0	Cv,Up	M	B,F,S,U,R,T		[11,15,28]
*Mormoops* sp.	1	0	0		T	G,R		[44]
* *Pteronotus fulvus*	12	1	8.3	C,Cv,Up	M	B,F,S,U,R,T	β	[15,21,28]
*Pteronotus gymnonotus*	2	0	0	Cv	Cr	F,R		[31]
* *Pteronotus mesoamericanus*	504	15	2.9	Cv,Up	Br,Cr,M,T	B,G,F,S,U,R,T	β	[15,21,28,29,31,44]
* *Pteronotus psilotis*	10	1	10	Up	M	S,U,R,T	α	[15,28]
*Pteronotus* sp.	1	0	0	Up	Br	S,U,R,T		[15]
*Noctilionidae*								
*Noctilio albiventris*	9	0	0	Cv,Up	B,Br,Cr	F,S,U,R,T		[15,28,31]
*Noctilio leporinus*	8	0	0	Up	B,Pa,T	G,F,S,U,R,T		[15,29,44]
*Phyllostomidae*								
*Ametrida centurio*	3	0	0	Up	Br	S,U,R,T		[15]
* *Anoura caudifer*	45	4	8.9	Up	B,Br,Pe	S,U,R,T		[15]
*Anoura cultrata*	5	0	0	Up	B,Cr	F,S,U,R,T		[15,31]
* *Anoura geoffroyi*	106	4	3.7	Up	B,Cr	F,S,U,R,T	α	[15,21]
*Anoura* sp.	4	0	0	Up	B,Br	S,U,R,T		[15]
								
*Artibeus anderseni*	15	0	0	Up	B	S,U,R,T		[15]
*Artibeus cinereus*	3	0	0	Up	Br	S,U,R,T		[15]
*Artibeus concolor*	4	0	0	Up	Br	S,U,R,T		[15]
*Artibeus fimbriatus*	4	0	0	Cv,Up	Br	S,U,R,T		[15,33]
*Artibeus glaucus*	1	0	0	Up	B	S,U,R,T		[15]
*Artibeus gnomus*	7	0	0	Up	Br,Pe	S,U,R,T		[15]
* *Artibeus jamaicensis*	754	14	1.8	Cv,Up	B,Cr,E,M,Pa,	B,G,F,S,U,R,T	α	[15,21,28,29,31]
* *Artibeus lituratus*	808	16	1.9	Cv,Up	B,Br,Cr,E,M,Pa,Pe	B,F,S,U,R,T	α,β	[15,21,28,29,30,31,33]
* *Artibeus obscurus*	61	1	1.6	Up	B,Br,E,Pe	F,S,U,R,T		[15,29,33]
* *Artibeus phaeotis*	149	6	4.0	A,Cv,Up	Cr,M,Pa	B,F,S,U,R,T	β	[15,21,28,29,31]
* *Artibeus planirostris*	235	1	0.4	Cv,Up	B,Br,Pe	S,U,R,T		[15,30,32]
*Artibeus* sp.	17	0	0	Up	B,Br,Pe	S,U,R,T		[15]
*Artibeus watsoni*	70	0	0	Cv,Ap	Cr,M,Pa	B,F,S,U,R,T		[15,21,29,31]
*Carollia benkeithi*	12	0	0	Up	B,Br	S,U,R,T		[15]
* *Carollia brevicauda*	222	6	2.7	Up	B,Br,E,Pe	F,S,U,R,T	α	[15,29]
* *Carollia castanea*	62	1	1.6	A,Up	B,Cr,E,Pa,Pe	B,F,S,U,R,T	α	[15,29,31]
*Carollia manu*	20	0	0	Up	B	S,U,R,T		[15]
* *Carollia perspicillata*	947	29	3.0	A,Cv,Up	B,Br,Cr,E,M,Pa,Pe,T	B,G,F,S,U,R,T	α	[15,21,28,29,30,31,33]
* *Carollia sowelli*	294	13	4.4	Cv,Up	Cr,M	B,F,S,U,R,T	α	[15,21,31]
*Carollia* sp.	53	0	0	Up	B,Br,Pe	S,U,R,T		[15,29]
*Carollia subrufa*	11	0	0	A,Cv,Up	Cr,M	S,U,R,T		[15,29]
*Centurio senex*	21	0	0	Cv,Up	M	B,F,S,U,R,T		[15,21]
*Chiroderma* sp.	1	0	0	Up	Pe	S,U,R,T		[15]
*Chiroderma trinitatum*	3	0	0	Up	Pe	S,U,R,T		[15]
*Chiroderma villosum*	3	0	0	Up	M	S,U,R,T		[15]
*Choeroniscus godmani*	20	0	0	Cv,Up	M	B,F,S,U,R,T		[15,21]
*Choeroniscus minor*	4	0	0	Up	B,Br	S,U,R,T		[15]
*Choeronycteris mexicana*	1	0	0	Up	M	S,U,R,T		[15]
*Chrotopterus auritus*	11	0	0	Cv,Up	Br,E,M	B,F,S,U,R,T		[15,21,29]
* *Desmodus rotundus*	210	3	1.4	A,Cv,Up	B,Br,Cr,M,Pa,Pe,T	B,G,F,S,U,R,T	α,β	[15,21,28,29,30,31,32,33,44,45]
*Diphylla ecaudata*	8	0	0	Up	Br,M	S,U,R,T		[15,30]
*Enchisthenes hartii*	5	0	0	Up	Cr,M,Pe	F,S,U,R,T		[15,29]
*Glossophaga commissarisi*	28	0	0	Cv,Up	Br,Cr,M	B,F,S,U,R,T		[15,21,29,31]
*Glossophaga morenoi*	4	0	0	Up	M	S,U,R,T		[15]
* *Glossophaga soricina*	439	6	1.3	A,Cv,Up	B,Br,Cr,M,Pa,Pe,T	B,G,F,S,U,R,T	α	[15,21,28,29,30,31,32,33,44]
*Glossophaga* sp.	5	0	0	Up	Br,M	S,U,R,T		[15]
*Hylonycteris underwoodi*	12	0	0	A,Up	Cr,M	F,S,U,R,T		[15,31]
*Lampronycteris brachyotis*	5	0	0	Up	B,Cr	F,S,U,R,T		[29,31]
*Lampronycteris* sp.	1	0	0	Up	Br	S,U,R,T		[15]
*Lonchophylla thomasi*	1	0	0	Up	Br	S,U,R,T		[15]
								
*Leptonycteris nivalis*	8	0	0	Up	M	B,F,S,U,R,T		[15,21]
*Leptonycteris yerbabuenae*	11	0	0	Up	M	B,F,S,U,R,T		[15,21]
* *Lichonycteris obscura*	2	2	100	Up	Br	S,U,R,T		[15]
*Lionycteris spurrelli*	1	0	0	Up	Br	S,U,R,T		[15]
*Lonchophylla robusta*	1	0	0		Cr	F		[29]
*Lonchophylla thomasi*	12	0	0	Up	B,Br	S,U,R,T		[15]
* *Lonchorhina aurita*	4	2	50	A,Cv,Up	Br,Cr,M	F,S,U,R,T	α	[15,21,29,31]
*Lophostoma* sp.	1	0	0	Up	Br	S,U,R,T		[15]
*Lophostoma brasiliense*	6	0	0	A,Up	B,Br,Cr,Pa	F,S,U,R,T		[15,29,31]
*Lophostoma silvicolum*	33	0	0	Up	B,Br,Cr,E,Pa	B,F,S,U,R,T		[15,29,31]
* *Mesophylla macconnelli*	13	1	7.6	Up	B,Br,E,Pe	F,S,U,R,T		[15,29]
*Micronycteris* sp.	1	0	0	Up	Pe	S,U,R,T		[15]
*Micronycteris hirsuta*	7	0	0	Up	Br,Pa	S,U,R,T		[15,29]
*Micronycteris megalotis*	1	0	0	Up	Br	S,U,R,T		[15]
*Micronycteris microtis*	11	0	0	A,Up	Br,Cr,M,Pa	B,F,S,U,R,T		[15,21,29,31]
*Micronycteris minuta*	2	0	0	Up	Br,Pa	B,F,S,U,R,T		[15,29]
*Micronycteris nicefori*	1	0	0	Up	Pe	S,U,R,T		[15]
*Micronycteris schmidtorum*	19	0	0	Cv,Up	M	B,F,S,U,R,T		[15,21]
*Micronycteris* sp.	1	0	0	Up	B	S,U,R,T		[15]
*Mimon cozumelae*	2	0	0	Cv,Up	M	B,F,S,U,R,T		[15,21]
*Mimon crenulatum*	79	0	0	Up	H,S,Sa,O,R,T	B,F,S,U,R,T		[15,21]
*Phylloderma stenops*	10	0	0	Cv,Up	B,Br,M,Pa	B,F,S,U,R,T		[15,21]
* *Phyllostomus discolor*	140	4	2.8	Cv,Up	Br,M,Pa	B,F,S,U,R,T	α	[15,21,29,30]
*Phyllostomus elongatus*	25	0	0	Up	B,Br,E,Pe	F,S,U,R,T		[15,29]
*Phyllostomus hastatus*	88	0	0	Up	B,Br,E,Pa,Pe,T	G,F,S,U,R,T		[15,29,30,44]
*Phyllostomus* sp.	2	0	0	Up	Br	S,U,R,T		[15]
*Platyrrhinus brachicephalus*	6	0	0	Up	B,E	F,S,U,R,T		[15,29]
*Platyrrhinus dorsalis*	1	0	0	Up	B	S,U,R,T		[15]
*Platyrrhinus fusciventris*	8	0	0	Up	Br	S,U,R,T		[15]
*Platyrrhinus helleri*	64	0	0	Cv,Up	Br,Cr,M,Pa,Pe	B,F,S,U,R,T		[15,21,29,31]
*Platyrrhinus infuscus*	2	0	0		E	F		[29]
* *Platyrrhinus lineatus*	22	1	4.5	Up	Br	F,S,U,R,T		[15,30,32,33]
*Platyrrhinus* sp.	19	0	0	Up	Br,Pe	S,U,R,T		[15]
*Platyrrhinus vittatus*	2	0	0	A	Cr	F,R		[31]
*Pygoderma bilabiatum*	1	0	0	Up	Br	S,U,R,T		[15]
*Rhinophylla fischerae*	8	0	0	Up	Br	S,U,R,T		[15]
*Rhinophylla pumilio*	47	0	0	Up	Br,E,Pe	F,S,U,R,T		[15,29]
*Rhinophylla* sp.	6	0	0	Up	Br	S,U,R,T		[15]
* *Sturnira erythromos*	16	2	12.5	Up	B	S,U,R,T		[15]
*Sturnira hondurensis*	278	0	0	A,Cv,Up	Cr,M	B,F,S,U,R,T		[15,21,28,31]
*Sturnira magna*	8	0	0	Up	E,Pe	F,S,U,R,T		[15,29]
*Sturnira mordax*	1	0	0	A	Cr	F,R		[31]
*Sturnira oporaphilum*	17	0	0	Up	B	S,U,R,T		[15]
* *Sturnira lilium*	160	8	5.0	Cv,Up	B,Br,Cr,E,Pe	B,F,S,U,R,T	α	[15,21,28,29,30,31,33]
*Sturnira* sp.	14	0	0	Up	Br,Pe	S,U,R,T		[15]
*Sturnira tildae*	12	0	0	Up	B,Br	S,U,R,T		[15]
*Tonatia bidens*	2	0	0	Up	Br,Pe	S,U,R,T		[15]
*Tonatia saurophila*	19	0	0	A,Cv,Up	Br,Cr,E,M,Pa,Pe	B,F,S,U,R,T		[15,21,29,31]
*Tonatia silvicola*	1	0	0	Up	Br	S,U,R,T		[15]
*Trachops cirrhosus*	30	0	0	Cv,Up	Br,Cr,E,M,Pa,Pe	B,F,S,U,R,T		[15,21,29,31]
*Trinycteris nicefori*	4	0	0	Up	B,Br	S,U,R,T		[15]
*Trinycteris* sp.	1	0	0	Up	Br	S,U,R,T		[15]
*Uroderma bilobatum*	75	0	0	A,Cv,Up	B,Br,Cr,E,M,Pa,Pe	B,F,S,U,R,T		[15,21,29,31]
*Uroderma magnirostrum*	1	0	0	Up	B	S,U,R,T		[15]
*Uroderma* sp.	1	0	0	Up	Pe	S,U,R,T		[15]
*Vampyressa bidens*	6	0	0	Up	B,E,Pe	S,U,R,T		[15,29]
*Vampyressa pussilla*	4	0	0	Cv,Up	Br	S,U,R,T		[15,30,33]
*Vampyressa* sp.	2	0	0	Up	Br	S,U,R,T		[15]
*Vampyressa thyone*	2	0	0	Up	B,Pa	B,F,S,U,R,T		[15,29]
*Vampyriscus nymphaea*	6	0	0	A	Cr	F,R		[31]
*Vampyrodes caraccioli*	7	0	0	Up	M,Pa,Pe	B,F,S,U,R,T		[15,29]
*Vampyrum spectrum*	3	0	0	Up	Br,M	S,U,R,T		[15]
*Thyropteridae*								
*Thyroptera discifera*	1	0	0	Up	Br	S,U,R,T		[15]
TOTAL	9371	346						

*: Species positive for coronavirus. ^†^ N: number of individuals examined. ^‡^ NP: number of positive individuals and the percentage (%) with respect to N. ^§^ CS: Collection Site (A: agricultural, C: cave, Cv: vegetation cover, Up: urban and peri-urban area) ^¶^ Country (A: Argentina, B: Bolivia, Br: Brazil, C: Canada, Cr: Costa Rica, E: Ecuador, U: United States, M: Mexico, Pa: Panama, Pe: Peru, T: Trinidad and Tobago) ^#^ Samples examined (G: throat fluids, F: feces, S: saliva, B: blood, U: urine, R: rectal fluids, T: tissues) ^††^ CoV (α: *Alphacoronavirus*, β: *Betacoronavirus*).

**Table 2:** Bat species in which experimental infection with coronavirus has been carried out in the Americas.

Bat Species	Cell Infection	Viral Isolation	Seroconversion	Coronavirus Adaptation	Coronavirus (+)	Reference
*Artibeus jamaicensis*	+	+	+	+	MERS-CoV	[46,47]
*Artibeus planirostris*				+	MERS-CoV	[47]
*Carollia perspicillata*	+			+	hCoV-EMC, MERS-CoV	[47,48]
*Desmodus rotundus*		+		+	BatCoV, MERS-CoV	[31,47,49]
*Eptesicus fuscus*				+	MERS-CoV	[47]
*Myotis lucifugus*	+			+	Myl-CoV, MERS-CoV	[43,47,49]
*Perimyotis subflavus*	+				hCoV-NL63	[40]
*Sacopterix bilineata*				+	MERS-CoV	[40]
*Tadarida brasiliensis*		+			Bat alphacoronavirus/UF-FWC/2016/3	[38]

## Supplementary Materials

The following are available online at https://www.mdpi.com/article/10.3390/v13071226/s1. Table S1: List of coronaviruses sequences and its presence in four families of bats.
