# Supplementary material for: Coronaviruses in Bats: A Review for the Americas
Source: Viruses. 2021 Jun 25;13(7):1226. doi: 10.3390/v13071226 (PMC8310043; doi:10.3390/v13071226)
Supplement: Supplementary file 1 [file viruses-13-01226-s001.zip › viruses-1226640-supplementary.pdf]

Suplementary material

Table S1. List of coronaviruses sequences and its presence in four families of bats. The numbers within the table indicate the number of bat species where coronavirus has been detected.

Clave	Virus	Phyllostomidae	Vespertilionidae	Molossidae	Mormoopidae
1	Appalachian Ridge CoV Strain-ARCoV.1	0	1	0	0
2	Appalachian Ridge CoV Strain-ARCoV.2	0	1	0	0
3	ARCoV	0	1	0	0
4	Bat alphacoronavirus/UF-FWC/2016/3	0	0	1	0
5	Bat Coronavirus/2173/2014/Cynomops planirostris/Brazil	0	0	1	0
6	Bat Coronavirus/2218/2014/Cynomops planirostris/Brazil	0	0	1	0
7	Bat Coronavirus/4292/2013/Desmodus rotundus/Brazil	1	0	0	0
8	Bat Coronavirus/4539/2013/Cynomops planirostris/Brazil	0	0	1	0
9	Bat Coronavirus/4702/2013/Cynomops abrasus/Brazil	0	0	1	0
10	Bat Coronavirus/4705/2013/Cynomops abrasus/Brazil	0	0	1	0
11	Bat Coronavirus/5026/2013/Cynomops abrasus/Brazil	0	0	1	0
12	Bat CoV/4620/2013/Glossophaga soricina/Brazil	1	0	0	0
13	BatCoV Pa306 CostaRica Glo sor 2014	1	0	0	0
14	BatCoV u21 CostaRica Art jam 2012	1	0	0	0
15	BatCoV/Art.lit./1816/BRA/2010	1	0	0	0
16	BatCoV/Art.lit./2064/BRA/2011	1	0	0	0
17	BatCoV/Art.lit./2294/BRA/2012	1	0	0	0
18	BatCoV/Car.per./1514/BRA/2011	1	0	0	0
19	BatCoV/Car.per./1516/BRA/2011	1	0	0	0
20	BatCoV/Car.per./1599/BRA/2012	1	0	0	0
21	BatCoV/Eum.gla./242/BRA/2013	0	0	1	0
22	BatCoV/Glo.sor./100/BRA/2014	1	0	0	0

23	BatCoV/Mol.ruf./63/BRA/2014	0	0	1	0
24	BatCoV/Mol.ruf./92/BRA/2013	0	0	1	0
25	BatCoV/Myo.nig./117/BRA/2013	0	1	0	0
26	BatCoV/Myo.rip./259/BRA/2013	0	1	0	0
27	BatCoV/POA/2012	0	0	2	0
28	BatCoV/POA/2012/105	0	0	2	0
29	BatCoV/POA/2012/127	0	0	2	0
30	BatCoV/POA/2012/132	0	0	2	0
31	BatCoV/POA/2012/143	0	0	2	0
32	BatCoV/POA/2012/144	0	0	2	0
33	BatCoV/POA/2012/44	0	0	2	0
34	BatCoV/POA/2012/94	0	0	2	0
35	BatCoV/POA/2012/96	0	0	2	0
36	BatCoV/POA/2012/B01	0	0	2	0
37	BatCoV/POA/2012/D09	0	0	2	0
38	BatCoV/POA/2012/E05	0	0	2	0
39	BatCoV/POA/2012/E07	0	0	2	0
40	BatCoV/POA/2012/E11	0	0	2	0
41	BatCoV/POA/2012/E9	0	0	2	0
42	BatCoV/POA/2012/G01	0	0	2	0
43	BatCoV/POA/2012/G03	0	0	2	0
44	BatCoV/Stu.lil./1573/BRA/2012	1	0	0	0
45	BatCoV/Stu.lil./1613/BRA/2012	1	0	0	0
46	BatCoV/Stu.lil./1617/BRA/2012	1	0	0	0
47	BatCoV309 CostaRica Cas cas 2014	1	0	0	0
48	BatCovDR/2007	1	0	0	0

49	BatCoV-M.rufus28/Brasil/2010	0	0	2	0
50	BatCoV-P.davyi49/México/2012	0	0	0	1
51	BatCoVT114 CostaRica Car per 2012	1	0	0	0
52	BtCoV/BRA100/Car per/BRA/2009	1	0	0	0
53	BtCoV/BRA114/Car bre/BRA/2009	1	0	0	0
54	BtCoV/BRA118/Car per/BRA/2009	1	0	0	0
55	BtCoV/BRA119/Car per/BRA/2009	1	0	0	0
56	BtCoV/BRA182/Mol ruf/BRA/2009	0	0	1	0
57	BtCoV/BRA344/Car bre/BRA/2009	2	0	0	0
58	BtCoV/BRAP103/Mol cur/BRA/2009	0	0	1	0
59	BtCoV/JP524/Art jam/PAN/2010	1	0	0	0
60	BtCoV/JP534/Art lit/PAN/2010	1	0	0	0
61	BtCoV/KCR15/Pte par/CRC/2012	0	0	0	1
62	BtCoV/KCR155/Pte par/CRC/2012	0	0	0	1
63	BtCoV/KCR180/Pte par/CRC/2012	0	0	0	1
64	BtCoV/KCR216/Car per/CRC/2010	1	0	0	0
65	BtCoV/KCR22/Pte par/CRC/2012	0	0	0	1
66	BtCoV/KCR230/Pte par/CRC/2010	0	0	0	1
67	BtCoV/KCR24/Ano geo/CRC/2010	1	0	0	0
68	BtCoV/KCR252/Car per/CRC/2010	1	0	0	0
69	BtCoV/KCR253/Car per/CRC/2010	1	0	0	0
70	BtCoV/KCR260/Car per/CRC/2012	1	0	0	0
71	BtCoV/KCR289/Ano geo/CRC/2010	1	0	0	0
72	BtCoV/KCR291/Ano geo/CRC/2010	1	0	0	0
73	BtCoV/KCR293/Ano geo/CRC/2010	1	0	0	0
74	BtCoV/KCR370/Pte par/CRC/2010	0	0	0	1

75	BtCoV/KCR372/Car per/CRC/2010	1	0	0	0
76	BtCoV/KCR90/Car per/CRC/2010	1	0	0	0
77	BtCoV/KCR91/Car per/CRC/2010	1	0	0	0
78	BtCoV/KP256/Art jam/PAN/2010	1	0	0	0
79	BtCoV/KP565/Art jam/PAN/2010	1	0	0	0
80	BtCoV/KP816/Phy dis/PAN/2011	1	0	0	0
81	BtCoV/KP817/Phy dis/PAN/2011	1	0	0	0
82	BtCoV/OCR11/Pte par/CRC/2011	0	0	0	1
83	BtCoV/PB675/Art lit/PAN/2011	1	0	0	0
84	Bt-CoV/Trinidad/1CO7B	1	0	0	0
85	Bt-CoV/Trinidad/1FY2B	1	0	0	0
86	BtCoV_BA19I	0	0	1	0
87	BtCoV_SJRP03I	0	0	1	0
88	BtCoV_SJRP03Li	0	0	1	0
89	BtCoV_SJRP03Lu	0	0	1	0
90	BtCoV_SJRP04I	0	0	1	0
91	BtCoV_SJRP05I	0	0	1	0
92	BtCoV_SJRP05Li	0	0	1	0
93	BtCoV_SJRP06Li	0	1	0	0
94	BtCoV_SJRP06Lu	0	1	0	0
95	BtCoV_SJRP08I	1	0	0	0
96	BtCoV_SJRP10Lu	1	0	0	0
97	BtCoV_SJRP16I	0	0	1	0
98	BtCoV_SJRP22I	1	0	0	0
99	BtCoV_SJRP44I	1	0	0	0
100	BtCoV_SJRP49I	0	0	1	0
101	BtCoV_SJRP52I	0	0	1	0

102	BtCoV_SJRP52Li	0	0	1	0
103	Mex_CoV-1	2	0	0	0
104	Mex_CoV-10	0	0	0	1
105	Mex_CoV-11	2	0	0	0
106	Mex_CoV-11a	1	0	0	0
107	Mex_CoV-11b	2	0	0	0
108	Mex_CoV-2	1	0	0	0
109	Mex_CoV-3	1	0	0	0
110	Mex_CoV-4	1	0	0	0
111	Mex_CoV-5a	1	0	0	0
112	Mex_CoV-5b	3	0	0	0
113	Mex_CoV-6	0	1	0	0
114	Mex_CoV-7	0	1	0	0
115	Mex_CoV-8	0	0	1	0
116	Mex_CoV-9	0	0	1	0
117	Myl-CoV-11-Bat10-T	0	1	0	0
118	Myl-CoV-11-Bat11-T	0	1	0	0
119	Myl-CoV-11-Bat12-T	0	1	0	0
120	Myl-CoV-11-Bat13-T	0	1	0	0
121	Myl-CoV-11-Bat14-T	0	1	0	0
122	Myl-CoV-11-Bat15-T	0	1	0	0
123	Myl-CoV-11-Bat16-T	0	1	0	0
124	Myl-CoV-11-Bat17-T	0	1	0	0
125	Myl-CoV-11-Bat1-C	0	1	0	0
126	Myl-CoV-11-Bat2-C	0	1	0	0
127	Myl-CoV-11-Bat3-C	0	1	0	0

128	Myl-CoV-11-Bat4-C	0	1	0	0
129	Myl-CoV-11-Bat5-C	0	1	0	0
130	Myl-CoV-11-Bat6-C	0	1	0	0
131	Myl-CoV-11-Bat7-C	0	1	0	0
132	Myl-CoV-11-Bat8-T	0	1	0	0
133	Myl-CoV-11-Bat9-T	0	1	0	0
134	Myl-CoV-12-Bat18-C	0	1	0	0
135	Myl-CoV-12-Bat19-C	0	1	0	0
136	Myl-CoV-12-Bat20-C	0	1	0	0
137	Myl-CoV-12-Bat21-C	0	1	0	0
138	Myl-CoV-12-Bat22-C	0	1	0	0
139	Myl-CoV-12-Bat23-C	0	1	0	0
140	Myl-CoV-12-Bat24-C	0	1	0	0
141	Myl-CoV-12-Bat25-C	0	1	0	0
142	Myl-CoV-12-Bat26-C	0	1	0	0
143	Myl-CoV-12-Bat27-C	0	1	0	0
144	Myl-CoV-12-Bat28-C	0	1	0	0
145	Myl-CoV-12-Bat29-C	0	1	0	0
146	Myl-CoV-12-Bat30-C	0	1	0	0
147	Myl-CoV-12-Bat31-C	0	1	0	0
148	Myl-CoV-12-Bat32-T	0	1	0	0
149	Myl-CoV-12-Bat33-T	0	1	0	0
150	Myl-CoV-12-Bat34-T	0	1	0	0
151	Myl-CoV-12-Bat35-T	0	1	0	0
152	Myl-CoV-12-Bat36-T	0	1	0	0
153	Myl-CoV-12-Bat37-T	0	1	0	0

154	Myl-CoV-12-Bat38-T	0	1	0	0
155	Myl-CoV-12-Bat39-T	0	1	0	0
156	Myl-CoV-12-Bat40-T	0	1	0	0
157	Myl-CoV-12-Bat41-T	0	1	0	0
158	New England CoV-NECoV	0	1	0	0
159	PREDICT_CoV-1	2	1	0	0
160	PREDICT_CoV-10	0	0	0	1
161	PREDICT_CoV-11	3	0	0	1
162	PREDICT_CoV-12	1	0	0	0
163	PREDICT_CoV-13	2	0	0	0
164	PREDICT_CoV-14	2	0	0	0
165	PREDICT_CoV-15	2	0	0	0
166	PREDICT_CoV-3	1	0	0	0
167	PREDICT_CoV-4	3	0	0	0
168	PREDICT_CoV-40	1	0	0	0
169	PREDICT_CoV-5	2	1	0	0
170	PREDICT_CoV-6	0	1	0	0
171	PREDICT_CoV-7	0	1	0	0
172	PREDICT_CoV-8	0	0	1	0
173	PREDICT_CoV-9	0	0	1	0
174	Scotophilus bat CoV 512	0	0	1	0
175	RM-Bat-CoV 09-07/2009 MV	0	2	0	0
176	RM-Bat-CoV 15/2006/ML	0	1	0	0
177	RM-Bat-CoV 429/2007 MV	0	1	0	0
178	RM-Bat-CoV 433/2007 MV	0	1	0	0
179	RM-Bat-CoV 453/2007 EF	0	2	0	0

180	RM-Bat-CoV 61/2007 EF	0	1	0	0
181	RM-BTCoV 11	0	1	0	0
182	RM-BTCoV 27	0	1	0	0
183	RM-BTCoV 3	0	1	0	0
184	RM-BTCoV 48	0	1	0	0
185	RM-BTCoV 6	0	1	0	0
186	RM-BTCoV 65	0	1	0	0
187	UNICAMP_bat_BR_14	0	0	1	0
